# Development of New Dual-Purpose Environmental Strategies for Effective Antibiotic Degradation Using Red Mud-Based Fenton Oxidation Catalysts

**DOI:** 10.3390/molecules30061298

**Published:** 2025-03-14

**Authors:** Yirong Zhao, Junxia Su, Bingqi Zhou, Fujie Li, Kang Mao, Muhammad Umair, Guopei Huang, Hua Zhang

**Affiliations:** 1 State Key Laboratory of Environmental Geochemistry, Institute of Geochemistry, Chinese Academy of Sciences, Guiyang 550081, China; 2 University of Chinese Academy of Sciences, Beijing 100049, China

**Keywords:** sulfamethoxazole, Fenton oxidation, red mud, resource utilization, sustainability

## Abstract

Mitigating antibiotic pollution is essential to combating antibiotic resistance, safeguarding ecosystems, ensuring food and water safety, and preserving the efficacy of antibiotics. Simultaneously, the comprehensive utilization of red mud is a key approach to reducing resource waste and ecological damage. This study investigates the use of iron components from red mud to prepare RM-nZVI/Ni for Fenton-like reactions, aimed at degrading antibiotics in water. By leveraging the inherent iron content in red mud, RM-nZVI/Ni was developed to achieve a dual-purpose environmental strategy: antibiotic degradation and solid waste resource recycling. The results demonstrate that 0.02 g/L of sulfamethoxazole (SMX) can be fully degraded within 15 min using 0.1 g/L of RM-nZVI/Ni and 6 mM of H_2_O_2_. Hydroxyl radicals (·OH) and Ni were identified as key contributors to SMX removal. Moreover, this system exhibits universality in degrading common antibiotics such as LFX, NFX, CIP, and TC. LC-MS analysis and DFT theoretical calculations indicate that the degradation byproducts are of lower toxicity or are non-toxic. Additionally, cost analysis suggests that RM-nZVI/Ni is a cost-effective and efficient catalyst. This research gives valuable insights into antibiotic degradation using red mud-based catalysts and offers guidance for expanding the high-value applications of red mud.

## 1. Introduction

Due to the critical role that they play in the prevention and treatment of diseases in both humans and animals, the global usage of antibiotics continues to rise. However, owing to incomplete degradation in vivo, a large portion of antibiotics are excreted into various environmental media through metabolic processes, either in the form of the parent compound or as transformed metabolites. This result may alter natural microbial communities and induce bacterial gene mutations in the environment. In this process, antibiotic resistance is one of the most serious ecological risks [1,2,3,4]. Investigations into the antibiotic content in China’s major rivers and sediments have revealed that sulfonamide antibiotics often exhibit relatively high concentrations in several rivers [5], finding themselves in the national-level antibiotic priority ranking list based on ecological risk assessment [6]. Currently, methods for removing sulfonamide antibiotics from the environment mainly include biological degradation [7], coagulation–precipitation [8], adsorption separation [9,10], catalytic degradation [11,12], and so on. Among these methods, lower-toxicity and non-toxic degradation products give catalytic degradation a clear advantage, especially the Fenton reaction.

As one of the advanced oxidation reactions (AOPs), aided by free radicals produced by the reaction of Fe^2+^ with H_2_O_2_, Fenton oxidation technology attacks organic pollutants and degrades their structures into low-toxicity or non-toxic small molecules. Nanometer zero-valent iron (nZVI) has high redox potential, high activity and a large surface area, and has been widely used in wastewater treatment in recent years [13,14]. Despite the confirmed significant role of nZVI in pollutant degradation systems, easy oxidization and particle aggregation are the leading limitations for long-term catalysis, antioxidation, and removal capacity performance. In previous studies, it was observed that bimetallic catalysts, as well as multimetallic ones, exhibit favorable physicochemical properties and synergistic effects. These characteristics play a crucial role in enhancing both catalytic activity and durability [15,16,17].

Red mud (RM) is a byproduct of the purification of alumina from bauxite. According to statistics, each ton of alumina produces 1–2 tons of red mud [18]. Due to its lack of viable utilization pathways, the current global stockpile of red mud has exceeded 3.5 billion tons, with an annual increase of approximately 200 million tons [19,20]. Given the vast stockpiles of red mud, the comprehensive utilization of this industrial waste has attracted widespread attention from research teams both domestically and internationally, particularly in the field of wastewater treatment [21,22,23,24]. Red mud, characterized by a large surface area and high porosity, is advantageous for reactant adsorption, making it an ideal catalyst carrier [25,26]. The catalytic performance of red mud in recent years’ studies employing red mud as a catalyst is summarized in Table 1. These catalysts are generally associated with issues such as high preparation costs, suboptimal catalytic performance, and elevated operating costs. Therefore, we consider that the abundant metal elements in red mud, such as iron and aluminum, offer promising potential for its application in Fenton systems [27,28,29,30,31,32]. Particularly, Ni doping can further promote system reactivity by forming a galvanic cell with it [33]. Furthermore, nZVI/Ni can facilitate the further decomposition of H_2_ generated during the Fenton process into hydrogen radicals (·H). This transformation enhances the catalytic efficiency by effectively targeting the pollutants [34]. Therefore, we consider the concept of “treating waste with waste” by utilizing iron-rich red mud to prepare a highly active, low-cost, and recyclable environmentally friendly iron-based multimetallic material to treat sulfamethoxazole (SMX), which poses significant environmental hazards.

In this study, we aim to effectively utilize the intrinsic elements of red mud to develop a novel, low-cost, recyclable, and highly efficient red mud-based catalyst, while investigating its influencing factors and underlying mechanisms. This work presents a new pathway for the valorization of red mud. Therefore, we developed a red mud-based multimetallic catalyst modified with Ni^0^ and nanoscale nZVI (denoted as RM-nZVI/Ni), which exhibited outstanding catalytic performance in enhancing the efficiency of the Fenton oxidation system. The additional iron source compensates for the iron demand in the Fenton reaction, while the red mud not only provides multiple metal elements but also utilizes its inherent properties to disperse the generated zero-valent metals, effectively preventing particle aggregation. A combination of RM-nZVI/Ni, H_2_O_2_ and sulfamethoxazole (SMX) establishes this Fenton reaction system. Subsequently, we investigated the feasibility of this system in degrading sulfamethoxazole (SMX) in water environments. We identified the main reactive species involved in SMX degradation and H_2_O_2_ activation, and discussed the key parameters (RM-nZVI/Ni dosage, H_2_O_2_ concentration, initial pH) and the impact of common anions in water (Cl^−^, HCO_3_^−^, H_2_PO_4_^−^, SO_4_^2−^, and NO_3_^−^) on SMX removal efficiency. Its effectiveness in practical application was assessed as the applicability of RM-nZVI/Ni in different media (ultrapure water, tap water, surface water, and distillery wastewater) and its ability to degrade other antibiotics. Finally, we explored the SMX degradation pathway through DFT calculations and LC-MS analysis, evaluating the toxicity changes in the system during the degradation process. The successful establishment of this reaction system provides a viable pathway for the comprehensive utilization of red mud and the efficient removal of antibiotics from wastewater.

## 2. Materials and Methods

### 2.1. Source of Red Mud

The red mud was collected from the red mud stockpile in Guizhou, China. Fresh red mud was stored for later use after air-drying and passing it through a 100-mesh sieve. The composition and parameters of the collected samples can be found in Figure S1.

### 2.2. Material Synthesis

**Figure 1:**
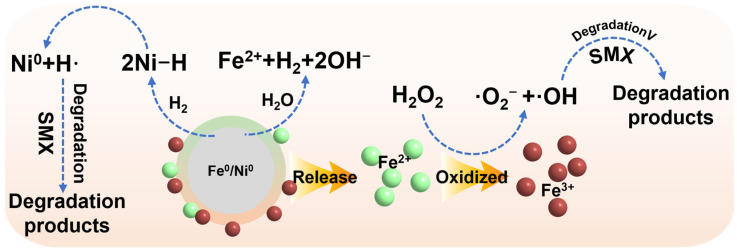
The degradation mechanism of SMX.

According to the iron content in red mud, achieving a 1:1 mass ratio between endogenous iron and the added iron source ensures an optimal balance. Additionally, even a trace amount of Ni doping enables the formation of a multimetallic system, significantly enhancing catalytic efficiency [50]. Therefore, to prepare RM-nZVI/Ni (Fe 1:1), 5 g of RM (ultrasonically dispersed evenly), 14.88 g of FeSO_4_·7H_2_O, and 0.2976 g of Ni(NO_3_)_2_·6H_2_O were dissolved in 300 mL of an ethanol–water solution (1:2, *v*/*v*) in a three-neck flask. The mixture was ultrasonicated for 30 min to ensure uniform dispersion. Then, in the N_2_ atmosphere, a 50 mL aqueous solution containing an excess of KBH_4_ was slowly added to the mixture solution under mechanical stirring for 60 min. The crude product was purified by filtering, washing, and drying overnight at 60 °C in a vacuum drying oven. The reaction equations involved are as follows, and the synthesis process is illustrated in Figure 1. The RM-nZVI/Ni (Fe 1:0.6) and RM-nZVI were prepared using a similar synthesis process. The specific addition ratios are provided in Table S1. The synthesis mechanism is illustrated in (1)–(3).

(1) $${\mathrm{Fe}}^{2+}+2{\mathrm{BH}}_{4}^{-}+6H_{2}O\;\text{→}{\mathrm{Fe}}^{0}+2{B\left(\mathrm{OH}\right)}_{3}+7H_{2}\text{↑}$$

(2) $${\mathrm{Ni}}^{2+}+2{\mathrm{BH}}_{4}^{-}+6H_{2}O\;\text{→}{\mathrm{Ni}}^{0}+2{B\left(\mathrm{OH}\right)}_{3}+7H_{2}\text{↑}$$

(3) $${\mathrm{Fe}}^{0}+{\mathrm{Ni}}^{2+}\text{→}{\mathrm{Fe}}^{2+}+{\mathrm{Ni}}^{0}$$

The chemical reagents used in this study are detailed in Text S1. The characterization methods of the catalysts are described in Text S2. The test methods of the antibiotics involved in the experiment and the toxicity prediction methods of the degradation products are shown in Text S3. The description of the Fenton oxidation experiment is shown in Text S4. And the theoretical calculation method of Density Functional Theory (DFT) is shown in Text S5.

## 3. Results and Discussion

### 3.1. Catalyst Characterization

**Figure 2:**
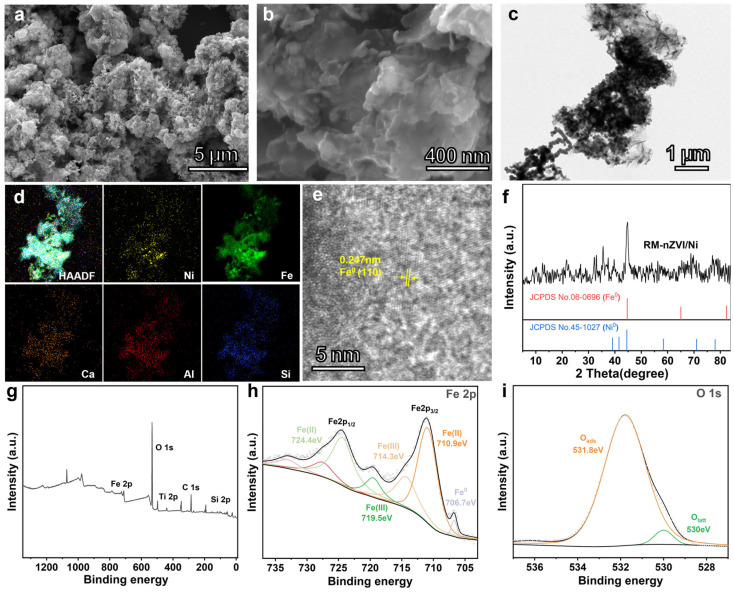
(**a**) Low-power SEM image of RM-nZVI/Ni; (**b**) high-power SEM image of RM-nZVI/Ni; (**c**) TEM image of RM-nZVI/Ni; (**d**) corresponding elemental mapping images and (**e**) HR-TEM image of TEM; (**f**) XRD pattern of RM-nZVI/Ni; (**g**) high-resolution full-spectrum XPS spectra of RM-nZVI/Ni; (**h**) high-resolution Fe 2p XPS spectra of RM-nZVI/Ni; and (**i**) high-resolution O 1s XPS spectra of RM-nZVI/Ni.

To observe the microstructure of the materials, field emission scanning electron microscope (SEM) and transmission electron microscopy (TEM) were used to characterize the untreated and modified RM and RM-nZVI/Ni. Figure S1 illustrates the irregular shape of the original RM surface. As shown in Figure 2a,b, the RM-nZVI/Ni showed nanosheets with some grains adhering to the smooth surface; this was perhaps the zero-valent metal growth on the red mud [51,52]. A TEM image revealing numerous black particles loaded on RM is shown in Figure 2c. Moreover, the elements Fe and Ni, and those inherently present in red mud, such as Ca, Al, and Si, were relatively uniformly distributed (Figure 2d), indicating the successful loading of nZVI and Ni^0^ on RM. As shown in Figure S2, the EDS elemental analysis demonstrated that the weight ratios of Fe, Ni, Al, and Si in the modified RM were 46.93%, 0.73%, 2.6%, and 16.12%, respectively, closely approximating the theoretical ratio of 50:1. Through XRD analysis, the crystallography and phase structure of the original RM sample (Figure S1) and the modified red mud (Figure 2f) were observed. A diffraction peak belonging to Fe^0^ appeared at 44.6°, corresponding to the (110) crystal plane of Fe^0^ (JCPDS No. 06-0696), consistent with the TEM result. The diffraction peak at 44.5° matched the standard diffraction pattern of Ni^0^ (JCPDS No.45-1027). Furthermore, compared to the original RM, the XRD spectrum of the reaction product lacked diffraction peaks of oxides, suggesting a partial reduction of the oxide components in red mud after the reaction. The XPS spectra revealed a peak of nZVI at 706.7 eV (Figure 2g,h) [53], indicating the successful reduction of Fe to nZVI. According to the XPS atomic percentage data (Table S3), the Ni atomic quantity was 0.4%, suggesting successful Ni attachment. However, there was no apparent peak in the XPS spectrum, which may be due to the low Ni content (<5%).

### 3.2. Assessment of the Performance of the RM-nZVI/Ni/H_2_O_2_ System

To ensure that the catalytic performance of the RM-nZVI/Ni/H_2_O_2_ material plays a primary role, different systems (RM-nZVI/Ni, H_2_O_2_, RM/H_2_O_2_, RM-nZVI/Ni/H_2_O_2_) were prepared at pH = 3 to investigate their degradation efficiency on 20 mg/L SMX. In the RM-nZVI/Ni and H_2_O_2_ systems, the SMX concentration remained nearly unchanged (Figure S2). For only the H_2_O_2_ process, the removal efficiency of SMX was only 1.01% after 30 min of reaction, indicating a weak oxidation capability of H_2_O_2_ alone towards SMX.

**Figure 3:**
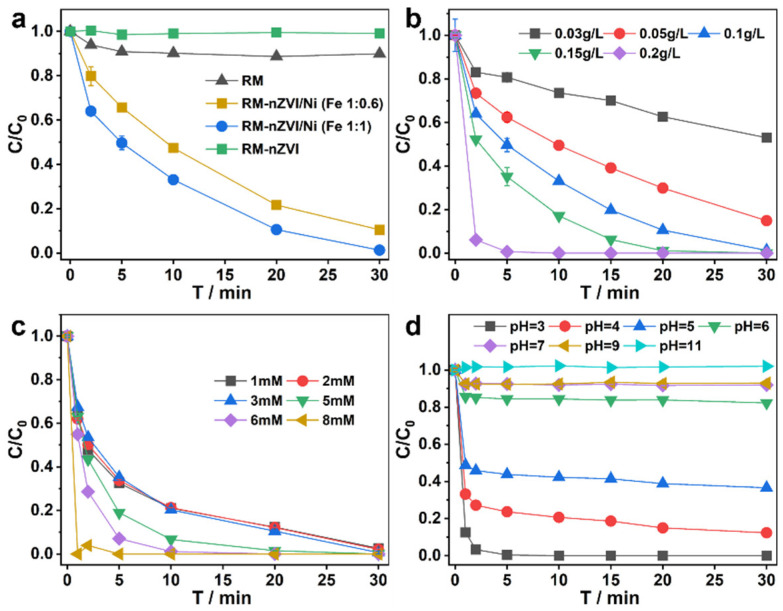
(**a**) Effect of different metal ratios on the degradation efficiency of SMX. (**b**–**d**) The influence of the key parameters, respectively, RM-nZVI/Ni dosage, H_2_O_2_ concentration, and initial pH.

Considering that the performance of the catalyst may be affected by the amount of iron doping, RM-nZVI/Ni catalysts with Fe content ratios of 1:0.6 and 1:1 were prepared. The Fe content was calculated based on the net Fe content in RM and FeSO_4_·7H_2_O, maintaining a constant Fe and Ni ratio. Additionally, to verify the necessity of adding the Fe-Ni bimetallic component, the RM-nZVI catalyst without Ni was prepared. As shown in Figure 3a, the untreated RM powder showed a removal efficiency of only 10.09% for SMX within 30 min under the action of H_2_O_2_. The degradation rates of SMX within 30 min using RM-nZVI/Ni (Fe 1:0.6), RM-nZVI/Ni (Fe 1:1), and RM-nZVI were 78.35%, 98.70%, and 2.29%, respectively. It can be seen that RM-nZVI/Ni (Fe 1:1) is more effective. The following reasons are proposed for this result. In the absence of Fe or Ni metals, the low catalytic efficiency is mainly due to the insufficient Fe content in RM to activate H_2_O_2_. Additionally, most metals in RM exist in a bound state, which is not conducive to metal valence conversion, leading to low catalytic efficiency when RM is used as a catalyst. Some studies have shown that the metal structure in composite materials can create multiple electron-rich regions, while the non-metallic part generates electron-deficient regions. This performance can accelerate the activation of H_2_O_2_ [50]. When metal elements are simultaneously reduced to the zero-valent state, there are two ways to improve catalytic efficiency. Firstly, the multi-metal co-catalysis mechanism enhances catalytic efficiency [54]. Secondly, the non-metallic components of the red mud can synergize with the metals to further boost catalytic performance. However, excessive metal doping may form a passivation layer on the material’s surface, and Fe aggregation can also negatively impact the catalytic reaction’s efficiency [55,56,57]. Therefore, the subsequent experiments in this study utilized RM-nZVI/Ni (Fe 1:1). Moreover, considering economic costs and environmental remediation, the costs are lower.

Additionally, we investigated the effects of catalyst dosage, oxidant dosage, and pH on the degradation efficiency of SMX. According to the experimental results, as the catalyst dosage increased within the range of 0.03–0.2 g/L, the reaction became faster (Figure 3b). H_2_O_2_ concentrations within the range of 1–8 mM resulted in a faster reaction at higher concentrations (Figure 3c). The degradation of SMX by the RM-nZVI/Ni/H_2_O_2_ system was effective under acidic conditions (pH = 3–5), and as acidity weakened, the degradation efficiency decreased. As shown in Figure 3c, within the pH range of 3–5, the RM-nZVI/Ni/H_2_O_2_ system effectively degraded SMX. However, the catalytic efficiency was completely inhibited in the pH range of 6–11 (Figure 3d). The optimal pH catalytic activity platform is suitable for most acidic wastewater environments, such as leachate, acidic coal mine wastewater, and metallurgical wastewater. Therefore, a system with a pH of 3, 0.1 g/L of RM-nZVI/Ni, and 6 mM H_2_O_2_ was used to initiate the reaction for subsequent experiments. Under optimized conditions, the RM-nZVI/Ni/H_2_O_2_ Fenton system demonstrated significantly higher removal performance, with 100% SMX degradation achieved in just 10 min. Compared to other studies, this system requires less H_2_O_2_ and catalyst, and the reaction time is shorter, making it more economically efficient [26,58].

### 3.3. Removal Mechanism of SMX in the Reaction System

#### 3.3.1. Identification of Key Reactive Oxygen Species (ROS) in the RM-nZVI/Ni/H_2_O_2_ System

**Figure 4:**
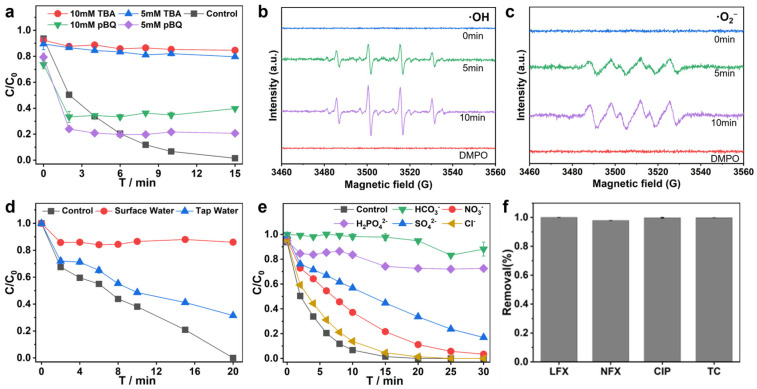
(**a**) Influence of tert-butyl alcohol (TBA) and p-benzoquinone (pBQ) on the degradation efficiency of SMX; (**b**,**c**) EPR spectra of DMPO-·O_2_^−^; (**d**) actual water sample verification experiment; (**e**) anion influence test; (**f**) degradation effect of other antibiotics within 4 min.

Based on previous studies, reactive species involved in H_2_O_2_-based Fenton reactions may include ·OH, ·O_2_^−^, or other active species [59,60]. EPR tests with DMPO as a radical scavenger confirmed the presence of both DMPO-·OH adducts in a 1:2:2:1 ratio (Figure 4b) and DMPO-·O_2_^−^ adducts (Figure 4c) in the RM-nZVI/Ni/H_2_O_2_ system, indicating that both ·OH and ·O_2_^−^ are involved in the catalysis. To further investigate the role of free radicals in the RM-nZVI/Ni/H_2_O_2_ catalytic system, free radical quenching experiments were conducted. Tert-Butanol (TBA) and p-Benzoquinone (pBQ) were used to scavenge ·OH and ·O_2_^−^ activity, respectively [61]. As shown in Figure 4a, the addition of TBA significantly decreased SMX removal efficiency, reaching only 15% within 15 min. While pBQ reduced the degradation efficiency of SMX, it still achieved around 70% removal within 15 min. These results suggest that ·OH plays a critical role in SMX degradation in this system, while ·O_2_^−^ contributes as a secondary factor.

#### 3.3.2. Changes in Metal Oxidation States in the RM-nZVI/Ni/H_2_O_2_ System

To further investigate the degradation mechanism of SMX in the RM-nZVI/Ni/H_2_O_2_ system, XPS analysis was performed on the materials before and after the oxidation reaction. It has been reported that the oxidation–reduction cycle between Ni^0^ and Ni^2+^ facilitates electron transfer, as well as the Fe^2+^/Fe^3+^ cycle [62]. As shown in Figure S4, the Fe2p_2/3_ spectrum reveals the presence of Fe^2+^, Fe^3+^, and Fe^0^ on the material’s surface prior to the reaction. After catalysis, the characteristic peaks of Fe^2+^ at 710.9 eV and Fe^0^ at 706.7 eV are significantly reduced, while the peak of Fe^3+^ at 714.3 eV is enhanced. This suggests that, as the reaction progresses, Fe^0^ and Fe^2+^ are oxidized to Fe^3+^ [63,64]. This decrease in the intensity of Fe^0^ and Fe^2+^ peaks, coupled with the increase in the Fe^3+^ peak area, indicates that Fe^0^ is partially consumed during the H_2_O_2_ activation process, contributing to the degradation of SMX.

### 3.4. Evaluation of the Practical Application Effect of the RM-nZVI/Ni/H_2_O_2_ System

#### 3.4.1. Impact of Inorganic Anions

Wastewater environments contain various inorganic anions, which may influence catalytic degradation efficiency [37]. To study their interference, the degradation efficiency was compared with a blank sample, as well as with the following anions: Cl^−^, HCO_3_^−^, H_2_PO_4_^−^, SO_4_^2−^, and NO_3_^−^ (Figure 4e). The removal efficiency in the presence of these anions was as follows: HCO_3_^−^ > H_2_PO_4_^−^ > SO_4_^2−^ > NO_3_^−^ > Cl^−^. In summary, the HCO_3_^−^ and H_2_PO_4_^−^ ions significantly inhibited SMX degradation, while the other anions maintained over 80% degradation efficiency, which aligns with previous reports. The inhibition of degradation by HCO_3_^−^ may be due to its role as a ·OH inhibitor [65], while H_2_PO_4_^−^ is a typical complexing agent that can form complexes with metal ions through coordination [66]. Therefore, it is crucial to address the influence of these two ions before applying the system in practical scenarios.

#### 3.4.2. Practical Evaluation

Water environments in nature vary significantly, and the effectiveness of AOPs in different water samples is a key factor in evaluating material performance. To assess this, we compared the removal efficiency of SMX by the RM-nZVI/Ni/H_2_O_2_ system in deionized water (ddw), tap water, and surface water. As shown in Figure 4d, the degradation efficiency in tap water was slightly lower than in ultrapure water, yet the system still removed 89.2% of the SMX within 60 min. This suggests that the RM-nZVI/Ni/H_2_O_2_ system demonstrates some anti-interference ability, making it suitable for practical applications. In surface water and heavily polluted distillery wastewater, the degradation efficiency dropped to 77.5% and 11.4%, respectively, likely due to the presence of interfering anions. These results indicate that the RM-nZVI/Ni/H_2_O_2_ system exhibits selectivity toward different water environments and highlight the importance of addressing unfavorable factors to enhance its practical application.

To evaluate the applicability of the RM-nZVI/Ni/H_2_O_2_ system for degrading different antibiotics, we tested four commonly used antibiotics: levofloxacin (LFX), norfloxacin (NFX), ciprofloxacin (CIP), and tetracycline hydrochloride (TC). As shown in Figure 4f, the degradation efficiency exceeded 98% within 4 min. Remarkably, LFX, CIP, and TC were already fully degraded within the first 2 min, falling below the detection limit. This performance significantly surpasses that reported in previous studies [67,68,69,70], highlighting the broad applicability of the RM-nZVI/Ni/H_2_O_2_ system for various antibiotics.

Upon the completion of the experiments, the RM-nZVI/Ni catalyst was easily separated from the wastewater using a commercial magnet thanks to its inherent magnetic properties (Figure S5). This allowed the treated water, free of organic pollutants, to proceed to the next processing step, while the recovered RM-nZVI/Ni catalyst was washed with clean water and dried for further use. The regenerated RM-nZVI/Ni catalyst can be reused with only a slight extension of the reaction time. Magnetism endows the catalyst with the advantages of easy recovery and handling, thereby ensuring environmental friendliness.

### 3.5. Degradation Pathway and Ecotoxicity Assessment

To gain a deeper understanding of the SMX degradation mechanism, it is crucial to identify the intermediate products and infer the primary degradation pathways in the RM-nZVI/Ni/H_2_O_2_ system. Using DFT calculations, we predicted the reactive sites on SMX and potential degradation products. These predicted intermediates were then confirmed through LC-MS analysis.

**Figure 5:**
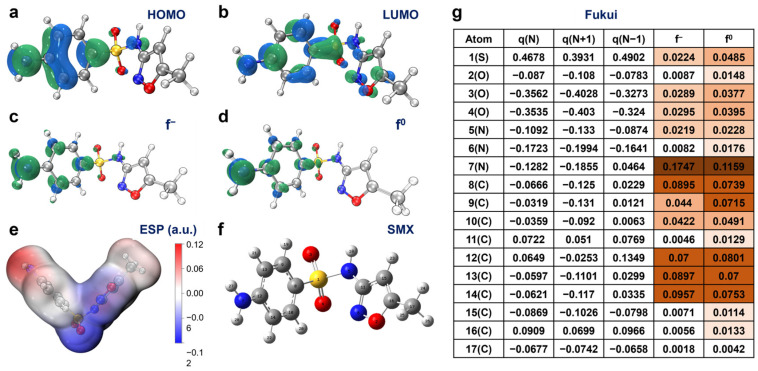
DFT. (**a**) HOMO, (**b**) LUMO, (**c**) *f^−^*, (**d**) *f*^0^, (**e**) electrostatic potential (ESP) map, (**f**) detailed atomic position of SMX, and (**g**) Fukui index.

The optimized molecular structure of SMX, as well as the distribution of HOMO and LUMO orbitals, are shown in Figure 5a,b. The HOMO-LUMO gap of 477.11 kJ/mol indicates a relatively small gap, suggesting high reactivity. The HOMO diagram highlights the sites most susceptible to attack by electrophilic radicals such as ·OH and ^1^O_2_ [71]. From the diagram, it can be observed that O3, O4, N5, N7, C8, C12, C13, and C14 are all susceptible sites for attack (Figure 5). However, the HOMO and LUMO alone cannot provide accurate information on the gain or loss of electrons at catalytic sites. Referring to Fukui indices and electrostatic potential (ESP) is therefore essential. Fukui indices (ƒ^−^ and ƒ^0^, representing electrophilic and radical attacks, respectively) are crucial indicators for estimating the reaction sites of pollutants (Figure 5g). The results of Fukui function mapping electron density isosurfaces and dual descriptor plots are depicted in Figure 5. In the RM-nZVI/Ni/H_2_O_2_ system, the degradation of SMX was primarily influenced by electrophilic free radicals, so we mainly considered the Fukui functions of electrophilic reactions (ƒ^−^) and free radical reactions (ƒ^0^). The higher these two values, the greater the reactivity. According to Figure 5g, N7 exhibited the highest reactivity, followed by C8, C9, C12, C13, and C14, but the other sites marked in this Figure also had the potential for reaction. Combined with Figure 4e, it can also be seen that the reactive sites were predominantly located in these positions.

**Figure 6:**
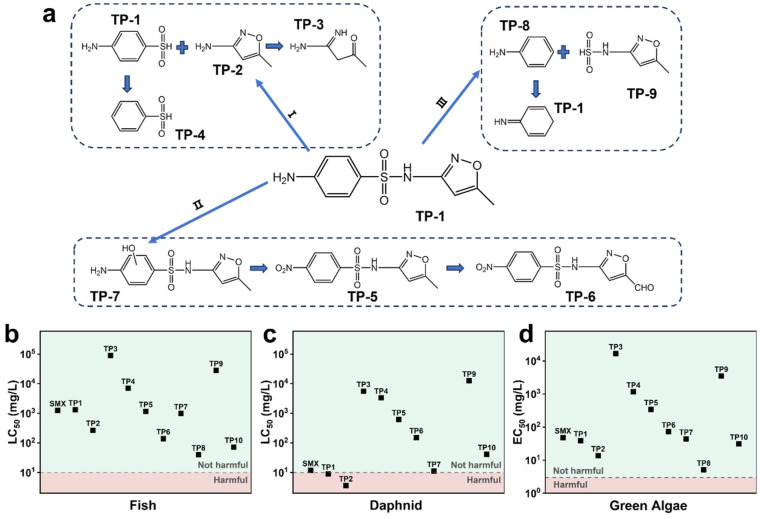
(**a**) The potential degradation pathway of SMX in the RM-nZVI/Ni/H_2_O_2_ system inferred from the LC-MS results; (**b**–**d**) ECOSAR ecological acute toxicity predictions of TPs for fish, daphnids, and green algae. LC_50_ represents the half-lethal concentration, and EC_50_ represents the half-effective concentration.

The chemical formulas, *m*/*z* values, and molecular structures of the detected degradation intermediates identified by LC-MS are provided in Table S4. Based on the detected intermediates and the results of the DFT calculations, we propose a potential degradation pathway of SMX in the RM-nZVI/Ni/H_2_O_2_ process, as illustrated in Figure 6a. These intermediates may undergo further oxidation by ROS during the reaction, leading to the formation of smaller products, ultimately transforming into CO_2_ and H_2_O.

According to the Ecological Structure–Activity Relationships (ECOSAR) model, the ecological toxicity of SMX and other identified transformation products (TPs) were predicted. Three representative aquatic organisms, namely fish, daphnid, and green algae, were used to assess their toxicity (Figure 6b–d). As shown in Figure 6, SMX exhibited no harm to fish and green algae, with only slight toxicity observed towards daphnids. The other TPs generated during the degradation process were non-toxic to fish and green algae. However, the TP3 from Pathway II and TP8 and TP9 from Pathway I exhibited greater toxicity to the daphnids compared to SMX. Nevertheless, TP3 could further transform into the non-toxic TP6 (Figure 6a). Therefore, based on our proposed degradation pathway, most of the TPs were non-toxic. Some highly toxic TPs generated in AOPs had unknown and unexplained degradation efficiencies. Therefore, in practical applications, it is essential to extend the overall AOP duration as much as possible to enhance the removal of these TPs.

### 3.6. Cost Estimation

The cost of preparing the RM-nZVI/Ni material is also a crucial factor in assessing its future application prospects. Hence, we investigated the market prices of the raw materials (per ton) used in its preparation and calculated the cost of producing 1 ton of the RM-nZVI/Ni material based on this information (ignoring factors such as electricity costs), as shown in Table 2. According to market prices, the cost of producing 1 ton of RM-nZVI/Ni is only 2701.14 USD. When compared to the price range of common antibiotic catalysts in the market, which is between 1378 and 3859 USD/ton, the preparation cost of the RM-nZVI/Ni material falls within a reasonable market range. Additionally, this material exhibits high degradation efficiency and requires minimal catalyst and oxidant usage, and its actual application cost is lower than that of many common catalysts in the market. Finally, considering the lower usage of the relatively expensive potassium borohydride in actual industrial production compared to laboratory settings, RM-nZVI/Ni still holds great potential for widespread application, even when considering economic costs.

## 4. Conclusions

In summary, the red mud-based bimetallic RM-nZVI/Ni material prepared in our study demonstrates superior performance in degrading SMX in wastewater through the activation of H_2_O_2_ in a Fenton-like reaction. Specifically, adding only small amounts of Fe and Ni can significantly enhance the catalytic performance of RM. The use of only 0.1 g/L RM-nZVI/Ni and 6 mM H_2_O_2_ shows a great degradation of 0.2 g/L SMX in 15 min. This process generates two types of free radicals, ·OH and ·O_2_^−^, with ·OH being the dominant factor. The material exhibits excellent broad-spectrum antibiotic degradation capabilities, showing high degradation rates for common antibiotics such as LFX, NFX, CIP, and TC, making it suitable for different antibiotic wastewater environments. Although preliminary studies have indicated that interfering anions (e.g., HCO_3_^−^ and H_2_PO_4_^−^) adversely affect the catalytic degradation efficiency, the underlying interference mechanisms and effective mitigation strategies should be further investigated. Through DFT calculations and LC-MS testing, possible degradation pathways of SMX were proposed. And an acute toxicity assessment predicted that most intermediate products are harmless to aquatic organisms. After cost accounting, the catalyst we prepared in the laboratory costs only 2701.14 USD/ton, within the market fluctuation range. With scale-up production, this cost is expected to decrease further. Considering cost-effectiveness, the RM-nZVI/Ni material in this study has advantages over most reported SMX degradation technologies. Therefore, the RM-nZVI/Ni material produced has the advantages of low cost, high efficiency, convenient separation and recovery, and the potential for solid waste reuse, making it highly suitable for practical applications. Future research will focus on conducting scale-up experiments under industrial conditions with various types of real wastewater to validate the treatment efficacy and economic feasibility of the RM-nZVI/Ni system in practical applications.

## Figures and Tables

**Table 1:** Comparison of the catalytic performance of red mud-based catalysts.

Pollutant	Concentration (mg/L)	Catalyst (g/L)	Oxidant	Concentration (mM)	pH	Time	Major ROS	Ref.
TC-HCl	20	0.2	PMS	1.0	3–11	30 min	SO_4_·^−^, ^1^O_2_	[35]
TC-HCl	40	0.3	PMS/vis	3.0	3–9	60 min	SO_4_·^−^	[36]
TC-HCl	20	0.2	PMS	1.0	3–11	60 min	SO_4_·^−^, ·OH, ^1^O_2,_ ·O_2_^−^	[36]
SMX	5.0	0.6	PDS	2.0	3–9	120 min	^1^O_2_	[26]
SDZ	20	0.2	PDS	2	3–9	20 min	SO_4_·^−^, ·O_2_^−^	[37]
Orange II	40	0.1	H_2_O_2_/vis	0.02	3–11	4 h	·OH	[28]
SMX	5.0	1.5	PMS	0.15	5–9	50 min	^1^O_2_	[38]
LOM-HCl	20	0.66	PMS	3.0	6.2	30 min	^1^O_2_	[39]
LVF	10	0.2	PMS	1.0	3–9	60 min	^1^O_2_	[40]
CIP	20	0.1	PMS	1.0	3–7	30 min	SO_4_·^−^, ·OH	[41]
CIP	20	0.5	H_2_O_2_	0.05	3–11	60 min	·OH, ^1^O_2,_ ·O_2_^−^	[42]
CIP	20	1.0	H_2_O_2_/vis/50 °C	0.02	3	180 min	·OH	[43]
Rh B	10	0.2	PMS	0.09	3–7	60 min	SO_4_·^−^, ·OH, ^1^O_2_	[44]
Rh B	20	2.0	H_2_O_2_/vis	0.0485	3–8.6	110 min	·OH, ^1^O_2,_ ·O_2_^−^	[45]
M-cresol	50	2.0	PMS	10	3–8	90 min	^1^O_2_	[46]
AMX	20	0.5	H_2_O_2_/vis	1	3–7	120 min	·OH, ·O_2_^−^	[47]
MB	50	0.1	H_2_O_2_/vis	0.02	3–9	15 min	·OH	[48]
	50	0.1	H_2_O_2_	0.02	3–9	30 min	·OH	
MB	40	0.1	H_2_O_2_	0.3	2–4	15 min	·OH, ·O_2_^−^	[49]

**Table 2:** Price estimation for preparing RM-nZVI/Ni material.

Raw Material	Market Price [72] (USD/ton)	Converted Price (USD/ton)
FeSO_4_·7H_2_O	35.83	28.94
N_2_	62.84	1.38
RM	0	0
Ni(NO_3_)_2_·6H_2_O	124.03	20.67
KBH_4_	12,127.56	2650.15
Cost calculation (USD/ton)	2701.14	

## Data Availability

Samples of the compounds are available from the authors.

## Supplementary Materials

The following supporting information can be downloaded at https://www.mdpi.com/article/10.3390/molecules30061298/s1, Text S1: Chemical reagents; Text S2: Catalyst characterization; Text S3: Analysis methods; Text S4: Catalytic performance testing; Text S5: Density Functional Theory (DFT); Table S1: The specific addition ratios for preparing RM-nZVI/Ni (Fe 1:0.6) and RM-nZVI; Table S2: HPLC and LC-MS analysis methods; Table S3: Atomic percent of XPS; Table S4: The chemical formulas, *m*/*z* values, and molecular structures of the detected degradation intermediates identified by LC-MS; Figure S1: Results of characterization of original red mud samples. (a,b): SEM; (c): XRD; (d): XRF; Figure S2: Quantitative result of EDS of RM-nZVI/Ni; Figure S3: Degradation efficiency of different systems (RM-nZVI/Ni, H_2_O_2_, RM/H_2_O_2_, RM-nZVI/Ni/H_2_O_2_) at pH = 3 on 20 ppm SMX; Figure S4: XPS Fe 2p before and after the response; Figure S5: The magnetic performance of the RM-nZVI/Ni catalyst. References [73,74,75] are cited in the supplementary materials.
