# Supplementary material for: Development of New Dual-Purpose Environmental Strategies for Effective Antibiotic Degradation Using Red Mud-Based Fenton Oxidation Catalysts
Source: Molecules. 2025 Mar 14;30(6):1298. doi: 10.3390/molecules30061298 (PMC11945986; doi:10.3390/molecules30061298)
Supplement: Supplementary file 1 [file molecules-30-01298-s001.zip › molecules-3519617-supplementary.pdf]

Supporting Information

Develop New Dual-Purpose Environmental Strategies for Effective Antibiotic Degradation Using Red Mud-Based Fenton Oxidation Catalysts

Yirong Zhao[a,b], Junxia Su[a], Bingqi Zhou[a,b], Fujie Li[a,b], Kang Mao[a], Muhammad Umair[a,b], Guopei Huang[a], and Hua Zhang[a]

[a] *State Key Laboratory of Environmental Geochemistry, Institute of Geochemistry, Chinese Academy of Sciences, Guiyang, 550081, China*

[b] *University of Chinese Academy of Sciences, Beijing 100049, China.*

Corresponding Email: sujunxia@mail.gyig.ac.cn (J. Su)

Number of text: 5

Number of table: 4

Number of figures:5

Content

Text.S1 Chemical Reagents.

Text.S2 Catalyst characterization.

Text.S3 Analysis methods.

Text.S4 Catalytic Performance Testing

Text.S5 Density Functional Theory (DFT)

Table.S1 The specific addition ratios for preparing RM-nZVI/Ni (Fe 1:0.6) and RM-nZVI.

Table.S2 HPLC and LC-MS analysis methods.

Table.S3 Atomic percent of XPS.

Table.S4 The chemical formulas, m/z values, and molecular structures of the detected degradation intermediates identified by LC-MS.

Fig.S1 Results of characterization of original red mud samples. a&b: SEM, c: XRD, d:XRF.

Fig.S2 quantitative result of EDS of RM-nZVI/Ni.

Fig.S3 Degradation efficiency of different systems (RM-nZVI/Ni, H_2O_2, RM/ H_2O_2, RM-nZVI/Ni/H_2O_2) at pH=3 on 20 ppm SMX.

Fig.S4 XPS Fe 2p of before and after the response.

Fig.S5 The magnetic performance of the RM-nZVI/Ni catalyst.

Text. S1 Chemical Reagents

Ferrous sulfate ($FeSO_4 \cdot 7H_2O$), Nickelous nitrate hexahydrate ($Ni(NO_3)_2 \cdot 6H_2O$), Potassium borohydride (KBH_4), Sodium hydroxide (NaOH), Hydrochloric acid (HCl), Methyl alcohol (MeOH, HPLC), Ethanol (EtOH), Hydrogen peroxide(H_2O_2, 30% w/v), Formic acid (HCOOH), Glacial acetic acid (CH_3COOH), Sulfamethoxazole (SMX), Sodium Chloride (NaCl), Sodium Bicarbonate ($NaHCO_3$), Sodium Dihydrogen Phosphate (NaH_2PO_4), Sodium sulfate (Na_2SO_4), Sodium nitrate($NaNO_3$), Tert-butyl alcohol (TBA), 5,5-dimethyl-1-pyrroline-*N*-oxide (DMPO). All the reagents were purchased from Aladdin and without further purification.

Text. S2 Catalyst characterization

The surface morphology of the original red mud sample and the synthesized RM-nZVI/Ni material were observed using a field emission scanning electron microscope (FESEM, JSM-7800F, Japan). Additionally, transmission electron microscopy (TEM) was employed to determine the structure and elemental distribution characteristics of the RM-nZVI/Ni material. The composition of the RM-nZVI/Ni phase was analyzed by X-ray powder diffraction (XRD, Bruker D2 PHASER, Germany). The surface composition and chemical states of the RM-nZVI/Ni material before and after the degradation reaction were confirmed through X-ray photoelectron spectroscopy (XPS, Thermo Scientific K-Alpha, USA). Electron paramagnetic resonance (EPR) signal spin-trapped by 5, 5-dimethyl-1-pyrroline N-oxide (DMPO), with an ESR spectrometer (ER200-SRC, Bruker Co., USA) was utilized to recognize the formation of radicals.

Text. S3 Analysis methods

To determine the residual concentration of SMX during the degradation process in this study, high-performance liquid chromatography (HPLC, 1800, Agilent, USA) equipped with a UV detector and a C18 column (4.6 mm × 150 mm) was used, with a mobile phase of 0.1% acetic acid aqueous solution and methanol. To determine the concentrations of other antibiotics, liquid chromatography-mass spectrometry (LC-MS, Ultimate 3000, ThermoFisher Scientific, USA) was employed with a mobile phase of 0.1% formic acid aqueous solution and 0.1% formic acid acetonitrile. The analysis methods for SMX and other antibiotics are listed in Table S1. Toxicity predictions for SMX and its transformation products (TPs) were conducted using the Toxicity Estimation Software Tool (T.E.S.T.) version 5.1.2 through quantitative structure-activity relationship (ECOSAR). According to the national standard GB/T 31270-2014 of China, the toxicity of the intermediates to fish, daphnid, and algae was used as an evaluation standard.

Text. S4 Catalytic Performance Testing

All experiments were conducted in 100 mL beakers. The beakers were placed on a horizontal shaker (OS-20Pro, JOANLAB, China) and the solution was stirred at a speed of 100 rpm at room temperature for 30 min. Initially, a 50 mL solution containing 20 mg/L of the target pollutant was prepared and placed in a 100 mL beaker. A measured amount of catalyst was added to the beaker, and the solution pH was adjusted to the desired value using 1.0 mol/L NaOH and 1.0 mol/L HCl. The pH value of the solution was measured using a pH meter (ST3100, OHAUS, China). After 30 min of adsorption, the required amount of H_2O_2 was added to the above water solution to activate the degradation reaction. At predefined time intervals, 1 mL of the post-reaction solution was taken from the beaker, added to excess tert-butanol, filtered through a 0.22 μm organic syringe filter, and transferred to a 2 mL liquid-phase sample bottle for subsequent analysis. The concentration of SMX was determined using high-performance liquid chromatography (HPLC). The concentrations of reaction products and other antibiotics were determined using liquid chromatography-mass spectrometry (LC-MS). For specific measurement methods, refer to Text.S3 and Table.S2. After the reaction, the RM-nZVI/Ni material was collected using a magnet.

Text. S5 Density Functional Theory (DFT)

All DFT calculations were performed with Gaussian16, A.03 software package [1]. The geometry optimization calculations were performed using the B3LYP functional and def2-SVP basis with the PCM solvation model for water, including Grimme dispersion corrections (GD3BJ). Then, the singlet point energy calculations were based on the B3LYP functional and a def2-TZVP basis with the SMD solvation model for water, including Grimme dispersion corrections (GD3BJ). The Fukui functions, ESP and HOMO/LUMO were achieved with the help of the Multiwfn 3.8(dev) program[2, 3].

Table. S1 The specific addition ratios for preparing RM-nZVI/Ni (Fe 1:0.6) and RM-nZVI

	**RM-nZVI/Ni (Fe 1:0.6)**	**RM-nZVI**
**RM**	3g	3g
**$FeSO_4 \cdot 7H_2O$**	14.88g	14.88g
**$Ni(NO_3)_2 \cdot 6H_2O$**	0.2976g	-

Table. S2 HPLC and LC-MS analysis methods.

<table>
<tr><td colspan="2">HPLC</td><td colspan="3">Chromatographic column: C18 4.6 mm × 150 mm
Eluents:2% Glacial acetic acid aqueous solution: methanol = 65:35
Flow rate: 1.0 mL/min; T: 6.5min; Wavelengths: 270nm</td></tr>
<tr><td rowspan="5">LC-MS</td><td>SMX and Extreme degradation product</td><td colspan="3">Chromatographic column: Accucore RP-MS 100×2.1mm
Eluents: (pos) A: 0.1% formic acid solution
B: 0.1% Acetonitrile formate solution
Spray voltage: +3.5kV;
(neg) A: 0.1% ammonium hydroxide
B: 0.1% Ammonia acetonitrile
Spray voltage: -3.0kV;
Flow rate: 0.3 mL/min
<table><tr><td>Time</td><td>%A</td><td>%B</td></tr><tr><td>0.0</td><td>95</td><td>5</td></tr><tr><td>0.5</td><td>95</td><td>5</td></tr><tr><td>12</td><td>5</td><td>95</td></tr><tr><td>16</td><td>5</td><td>95</td></tr><tr><td>17</td><td>95</td><td>5</td></tr><tr><td>20</td><td>95</td><td>5</td></tr></table></td></tr>
<tr><td>LFX</td><td>24eV</td><td rowspan="4">Chromatographic column: Accucore RP-MS 100×2.1mm
A: 0.1% formic acid solution;
B: 0.1% Acetonitrile formate solution
Spray voltage: +3.5kV; Flow rate: 0.3 mL/min
<table><tr><td>Time</td><td>%A</td><td>%B</td></tr><tr><td>0.0</td><td>85</td><td>15</td></tr><tr><td>0.3</td><td>85</td><td>15</td></tr><tr><td>7.0</td><td>5</td><td>95</td></tr><tr><td>9.0</td><td>5</td><td>95</td></tr><tr><td>9.5</td><td>85</td><td>15</td></tr><tr><td>12.0</td><td>85</td><td>15</td></tr></table></td></tr>
<tr><td>NFX</td><td>19eV</td></tr>
<tr><td>CIP</td><td>45eV</td></tr>
<tr><td>TC</td><td>22eV</td></tr>
</table>

Table. S3 Atomic percent of XPS

Name	Before Atomic %	After Atomic %
Al2p	3.9	6.79
C1s	25.89	28.53
Fe2p	5.28	13.03
Ni2p	0.43	0.97
O1s	61.18	46.15
Si2p	2.93	4.01
Ti2p	0.4	0.52

Table. S4 The chemical formulas, m/z values, and molecular structures of the detected degradation intermediates identified by LC-MS

Products	m/z	Ion Formula	Structure
SMX	253.1	$C_{10}H_{11}N_3O_3S$	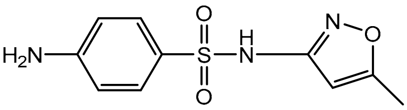
TP1	157.0	$C_6H_7NO_2S$	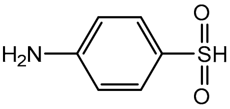
TP2	98.0	$C_4H_6N_2O$	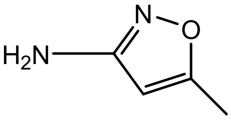
TP3	100.1	$C_4H_8N_2O$	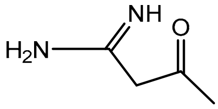
TP4	142.0	$C_6H_6O_2S$	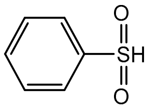
TP5	283.0	$C_{10}H_9N_3O_5S$	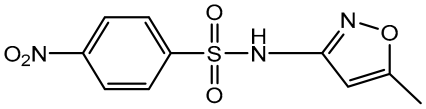
TP6	297.0	$C_{10}H_7N_3O_6S$	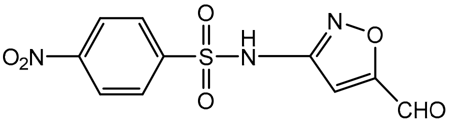
TP7	285.1	$C_{11}H_{15}N_3O_4S$	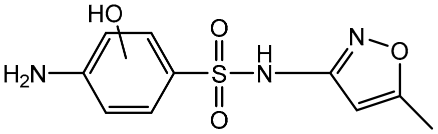
TP8	93.1	C_6H_7N	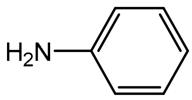
TP9	162.0	$C_4H_6N_2O_3S$	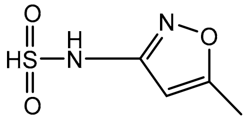
TP10	93.1	C_6H_7N	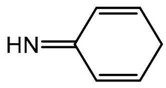

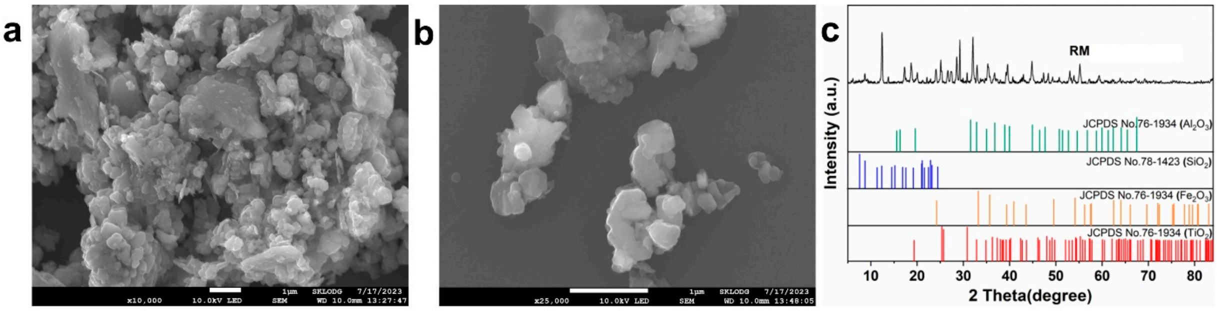

d

Oxide species	SiO_2	Al_2O_3	Fe_2O_3	MgO	CaO	Na_2O	K_2O	MnO	P_2O_5	TiO_2	SO_3	LOI	SUM
%	14.02	19.88	20.13	2.12	19.71	3.48	1.07	0.049	0.266	4.700	1.800	12.22	99.44

Fig. S1 Results of characterization of original red mud samples. a&b: SEM, c: XRD, d:XRF.

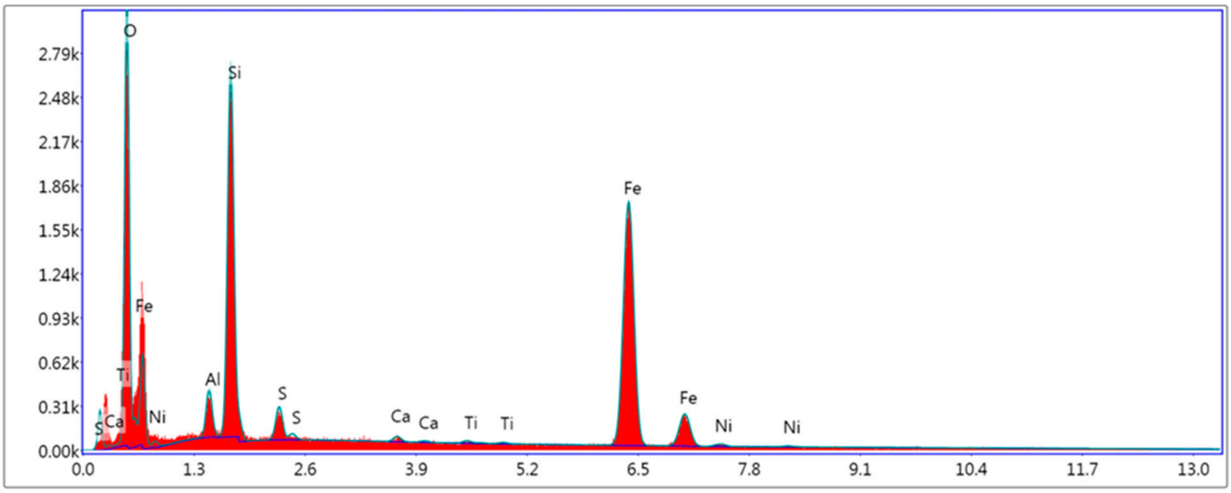

Fig.S2 quantitative result of EDS of RM-nZVI/Ni.

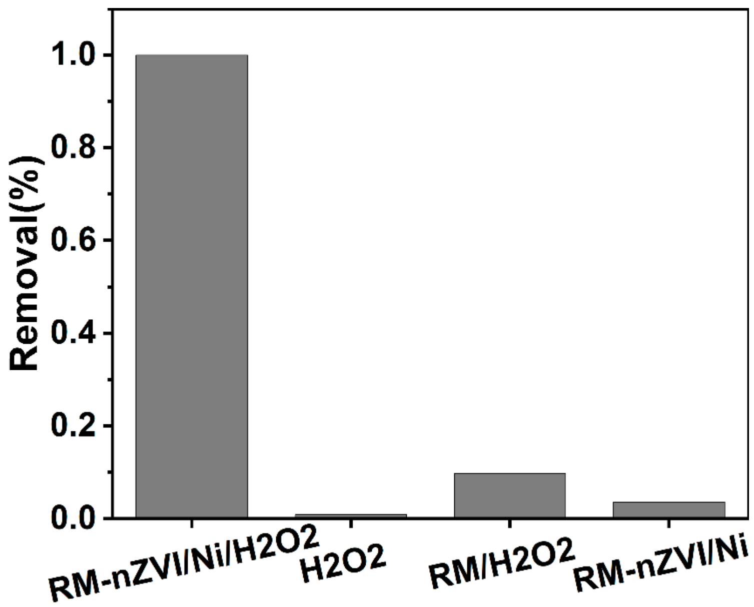

Fig.S3 Degradation efficiency of different systems (RM-nZVI/Ni, H_2O_2, RM/H_2O_2, RM-nZVI/Ni/H_2O_2) at pH=3 on 20 ppm SMX

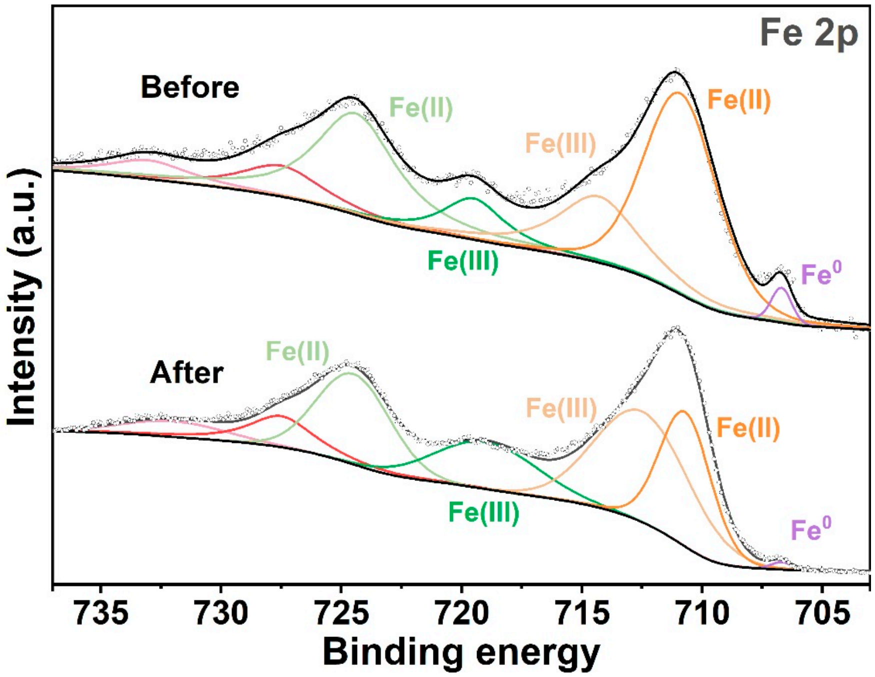

Fig.S4 XPS Fe 2p of before and after the response

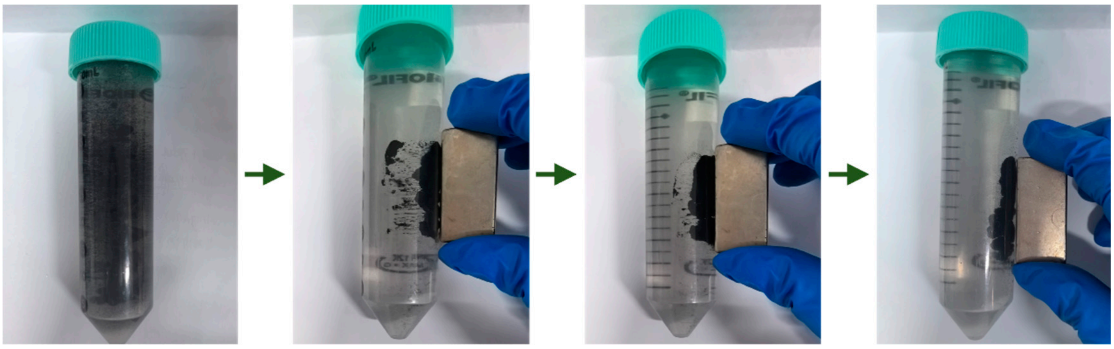

Fig.S5 The magnetic performance of the RM-nZVI/Ni catalyst

Renfences

[1] M.J. Frisch, G.W. Trucks, H.B. Schlegel, G.E. Scuseria, M.A. Robb, J.R. Cheeseman, G. Scalmani, V. Barone, G.A. Petersson, H. Nakatsuji, X. Li, M. Caricato, A.V. Marenich, J. Bloino, B.G. Janesko, R. Gomperts, B. Mennucci, H.P. Hratchian, J.V. Ortiz, A.F. Izmaylov, J.L. Sonnenberg, Williams, F. Ding, F. Lipparini, F. Egidi, J. Goings, B. Peng, A. Petrone, T. Henderson, D. Ranasinghe, V.G. Zakrzewski, J. Gao, N. Rega, G. Zheng, W. Liang, M. Hada, M. Ehara, K. Toyota, R. Fukuda, J. Hasegawa, M. Ishida, T. Nakajima, Y. Honda, O. Kitao, H. Nakai, T. Vreven, K. Throssell, J.A. Montgomery Jr., J.E. Peralta, F. Ogliaro, M.J. Bearpark, J.J. Heyd, E.N. Brothers, K.N. Kudin, V.N. Staroverov, T.A. Keith, R. Kobayashi, J. Normand, K. Raghavachari, A.P. Rendell, J.C. Burant, S.S. Iyengar, J. Tomasi, M. Cossi, J.M. Millam, M. Klene, C. Adamo, R. Cammi, J.W. Ochterski, R.L. Martin, K. Morokuma, O. Farkas, J.B. Foresman, D.J. Fox, Gaussian 16 Rev. A.03, Wallingford, CT, 2016.https://gaussian.com.

[2] T. Lu, F. Chen, Multiwfn: A multifunctional wavefunction analyzer, J. Comput. Chem., 33 (2012).https://doi.org/10.1002/jcc.22885.

[3] T. Lu, Q. Chen, Realization of conceptual density functional theory and information-theoretic approach in multiwfn program, CDFT2022, 631-647.https://doi.org/10.1002/9783527829941.ch31.
